# New test for sensitivity evaluation of *Trichomonas vaginalis* to nitroimidazoles and nitrofurans *in vitro*

**Published:** 2011-01-10

**Authors:** NS Makhlay

**Affiliations:** 1 Laboratory of Biopreparations, St.Petersburg Pasteur Institute, St.Petersburg 197101, Russia

## 

*Trichomonas vaginalis* (Tv) is a causative agent of worldwide spread sexually transmitted disease, particularly affecting underdeveloped all segments of the population. Metronidazole is by far the most prescribed drug for the treatment of trichomoniasis. However, the appearance of drug resistant has led to the appearance of new drug alternatives. That is why the development of reliable and standard test for the evaluation clinical isolates sensitivity is necessary for routine laboratory practice. Traditionally used for this purpose the method of serial dilutions is laborious and time consuming.

A simple and practical method can be used for screening of sensitivity of Tv to anti-trichomonas drugs. This test contains of nutrient medium, special device for microscopic study and 8-well strip with drugs in fixed concentration which was previously determined. Using ten laboratory strains Tv it was found that minimal inhibitory concentrations (MIC’s) were the following (µg/ml): metronidazole (5), secnidazole (2,5), tinidazole (2,5), nimorazole (2,5), ornidazole (2,5), clotrimazole (15), nifuratel (1,25). Viability definition of Tv was performed using trypan blue stain and subculturing on a medium without any drugs. The scheme of assay was the following: poured into eight strip wells nutrient medium, inoculum cells trichomonads added and do microscopic examination after 48 hours of incubation under 37°C. The microscopic technique is based on the use of a special device consisting of eight micro chambers which allows examining eight probes at a one operation. 99 patients who had been confirmed of having chronical trichomoniasis were examined with this test. The test sensitivity to metronidazole, secnidazole, tinidazole, nimorazole, ornidazole, clotrimazole and nifuratel was performed as described earlier.

Results of sensitivity test with clinical samples were divided into three groups: resistance (R) - maintaining the viability of 50% or more trichomonads cells, sensitivity I (SI) - presence of 25% or less viable cells, sensitivity II (SII) – all cells lysis. The results showed the following sensitivity samples distribution: metronidazole - 23 (R), 47 (SI), 29 (SII), tinidazole - 58 (R), 30 (SI), 11 (SII), nimorazol - 71 (R), 19 (SI), 9 (SII), ornidazole - 51 (R), 41 (SI), 7 (SII), seknidazol - 57 (R), 28 (SI), 17 (SII), clotrimazole - 60 (R), 32 (SI), 7 (SII), nifuratel - 32 (R), 52 (SI), 15 (SII). One strain possessed multiple resistance.

Thus, the frequency of strains detection resistant to the action of drugs quite high, that argues the need to determine it "in vitro" for the choice of subsequent therapy.

